# Association between Non-Suicidal Self-Injury, Parents and Peers Related Loneliness, and Attitude Towards Aloneness in Flemish Adolescents: An Empirical Note

**DOI:** 10.5334/pb.385

**Published:** 2018-02-20

**Authors:** Amarendra Gandhi, Koen Luyckx, Luc Goossens, Shubhada Maitra, Laurence Claes

**Affiliations:** 1 Faculty of Psychology and Educational Sciences, KU Leuven, BE; 2 Centre for Health and Mental Health, Tata Institute of Social Sciences, IN; 3 Faculty of Medicine and Health Sciences (CAPRI), University of Antwerp, BE

**Keywords:** Non-Suicidal Self-Injury, Parent-related loneliness, Peer-related loneliness, Attitude towards aloneness, Adolescents

## Abstract

Loneliness and attitude towards aloneness have been shown to be associated to depression, anxiety, and other psychiatric disorders in adolescents and they may also increase the vulnerability to Non-Suicidal Self-Injury (NSSI). Therefore, the present study investigated the association between lifetime prevalence and functions of NSSI, parent- and peer-related loneliness, and attitude towards aloneness (positive and negative). Data regarding NSSI, loneliness, and attitude towards aloneness were collected from a sample of 401 high school students from three different high schools located in the Dutch-speaking part of Belgium. Lifetime prevalence of NSSI was found to be 16.5%. Females reported a higher lifetime prevalence of NSSI than males. Higher mean scores for parent-, peer-related loneliness, and positive attitude (i.e., affinity) towards aloneness was observed in adolescents with lifetime NSSI as compared to adolescents without a history of NSSI. Finally, a positive correlation between self-related (i.e., automatic) functions of NSSI and parent- and peer-related loneliness and a positive attitude towards aloneness was also observed.

## Introduction

Non-Suicidal Self-Injury (NSSI) is defined as the deliberate destruction of one’s body tissue without an intention to die (Nock, 2009). The most common forms of NSSI include scratching, cutting, head-banging, and burning. In a recent meta-analysis of 119 studies, Swannell and colleagues (2014) reported an international pooled prevalence of NSSI among adolescents to be around 17.2%, among young adults to be around 13.4%, and among adults to be around 5.5% adults. In Belgium, the prevalence of NSSI has been shown to range from 13.7%–26.5% in adolescents (Baetens, Claes, Muehlenkamp, Grietens, & Onghena, 2011; Claes, Luyckx, & Bijttebier, 2014). NSSI has been shown to be linked with developmental issues like disturbances in identity formation (Gandhi et al., 2017) and scholastic issues (Kiekens et al., 2016). Chronic engagement in NSSI has been shown to be strongly associated with various mental health issues like depression, anxiety disorder, substance use disorder, borderline personality disorder, and eating disorders (Nock, 2009). The history, frequency, and number of different methods of NSSI have also been shown to be important predictors of suicide attempts (Victor and Klonsky, 2014).

Although NSSI seems to be related to significant distress, the majority of adolescents engaging in NSSI do not seek help for it (Muehlenkamp, Walsh, & McDade, 2010). Therefore, NSSI is being increasingly identified as an important mental health concern, especially in adolescents. Extant research has identified NSSI as an outcome of a complex interaction between interpersonal [e.g., poor attachment (You, Lin, & Leung, 2015), bullying, and abuse history (Garisch & Wilson, 2015)] and intrapersonal factors [e.g., alexithymia, depression, anxiety, impulsivity, substance abuse, and sexuality (Garisch & Wilson, 2015)]. However, to further increase our understanding of adolescent NSSI, stressors that are particularly relevant during this period of life should be investigated.

Loneliness is one of the important stressors in adolescents. In fact, about 80% of adolescents’ report experiencing loneliness at least some of the time as compared to the 40% of adults (Hawkley & Cacioppo, 2010; Heinrich & Gullone, 2006). An extensive review by Qualter and colleagues (2010) has identified a number of negative mental health outcomes and psychiatric disorders associated with loneliness. The negative mental health outcomes associated with loneliness include issues like low self-esteem, shyness, neuroticism, social withdrawal, poor academic performance, and juvenile delinquency (Qualter et al., 2010). Persistent loneliness has also been shown to be connected to psychiatric conditions like personality disorders (avoidant, borderline, and dependent personality disorder; Qualter et al., 2010), depressive symptoms (Vanheule, Desmet, Groenvynck, Rosseel, & Fontaine, 2008), and social anxiety (Caplan, 2006). Importance of different relationships change over different phases of life and if these relationship needs are not met, individuals may develop loneliness with respect to the that relationship (Lasgaard et al., 2004). Although the relevance of peer relationships gradually increases during adolescence, the significant of the relationship with parents does not diminish (Hazel, Oppenheimer, Technow, Young, & Hankin, 2014). Consequently, parent and peers related loneliness can both contribute to increased vulnerability to psychopathology (Lasgaard et al., 2004).

The brief review presented above not only emphasizes the importance of loneliness in adolescents but also highlights the need of investigating the association between NSSI and loneliness. Yet, as far as we are aware, the studies by Giletta et al., (2012) and Lasgaard et al., (2011) are the only ones that have explored the relation between loneliness and NSSI in community samples. Both studies found higher parent-related loneliness in adolescents with NSSI. However, no differences in peer-related loneliness were observed in adolescents with and without NSSI. Loneliness has also been linked to the behavioral motivations (i.e., functions) for engaging in NSSI. The functional model of NSSI focuses on the purpose of NSSI and the factors that reinforce self-injury (Nock, 2009). According to Nock (2009), the functions of NSSI can be grouped into four categories: positive social (e.g., to get support or attention from others); negative social (e.g., to escape social situations); positive automatic (e.g., to feel something); and negative automatic (e.g., to avoid negative affect). Nock and Prinstein (2005) hypothesized that as loneliness is an interpersonal concern, it may be associated with the social function of NSSI (e.g., facilitation of help-seeking or escape from undesired social situations). However, in a clinical adolescent sample, they did not observe any relation between loneliness and both automatic and social functions of NSSI.

Like loneliness, attitude towards aloneness has also been identified as a significant factor that can influence development in adolescents. Aloneness is defined as the physical and neutral state of being on one’s own and attitude towards aloneness is the positive or negative evaluation of the state of aloneness (Galanaki, 2013). Extreme affinity or aversion to aloneness has been shown to be associated with negative mental health issues (Wang et al., 2013). Goossens and Marcoen, (1999) demonstrated that adolescents with higher positive aloneness were more likely to suffer from social anxiety and depression. This counterintuitive association can be explained by the item structure of the positive attitude towards aloneness subscale of the Loneliness and Aloneness Scale for Children and Adolescents (LACA) used in the study by Goossens & Marcoen, (1999). According to these authors, the subscale -positive attitude towards aloneness captures reactive rather than active affinity to aloneness (Galanaki, 2013; Goossens & Marcoen, 1999). That is, adolescents demonstrating positive attitude towards aloneness, may spend time alone not because they like to do so but because they want to avoid the company of others. On the other hand, negative attitude towards aloneness can intensify feelings of loneliness, boredom, and negative feelings (Marcoen, Goossens, & Caes, 1987). In sum, although attitude towards aloneness may be an important factor associated with mental health in adolescents, its relation with NSSI has remained unexplored.

The present exploratory study had two objectives. First, we explored whether adolescents with and without NSSI had significant mean differences in the scores of parents- and peer-related loneliness and attitude towards aloneness. Based on the existing literature (Giletta et al., 2012; Lasgaard et al., 2011), we expected adolescents with NSSI to have higher mean scores on parents- and peer loneliness. As no studies have explored the association between NSSI and attitudes towards loneliness yet, we did not formulate a hypothesis regarding this relationship. Second, we explored the correlation between the functions of NSSI and parents-and peers- related loneliness and attitudes towards aloneness. As loneliness can be experienced as an aversive emotional state and NSSI may serve as a behavior to manage the distress associated with loneliness, we expected both parents- and peers-related loneliness to be positively associated with the automatic (i.e., intrapersonal) functions of NSSI. Again, no specific expectation could be formulated regarding functions of NSSI and the attitudes towards aloneness because of the lack of previous research.

## Methods

### Participants and procedure

Data were collected in three different high schools located in the Dutch-speaking part of Belgium. Four hundred and one participants (51.5% females; Grades 10–12; 97.5% with Belgian nationality) were recruited by convenience sampling. Mean age was 16.6 years (*SD* = 0.96; range = 14–19 years). Informed consent letters were provided to the parents of the students about two weeks before the day of data collection. Students were permitted to participate in the study only if they had parental consent. The data collection procedure was completed during the school hours. Students were provided with an envelope including assent form and the questionnaires and they were requested to return the completed forms in a sealed envelope. The researchers were present throughout the data collection process to answer questions regarding any aspect of the study. Contact details of mental health services in Flanders were also provided if students required assistance. The study was approved by the Institutional Review Board at the researchers’ university.

### Measures

Lifetime NSSI was assessed by means of a single YES/NO question (“Have you ever injured yourself on purpose without an intention to die?”). The use of single-item measure is common in the NSSI literature and a review by Muehlenkamp, Claes, Havertape, and Plener (2012) indicated that the prevalence of NSSI does not significantly differ across the different methods used (i.e., single-item or checklist method). In case participants answered this question affirmatively, they were asked to indicate the degree to which they endorsed 18 functions of NSSI (from the Self-Injurious Questionnaire-Treatment Related; Claes & Vandereycken, 2007). The items associated with the functions of NSSI (see Table 1 below for items included in the scale) were measured on a five-point Likert scale ranging from 1 (*not applicable*) to 5 (*very applicable*).

**Table 1:** Factor structure for the functions of NSSI scale as proposed by Gandhi et al., (2016). The third section represents the items not considered for calculating the factor scores as they had factor loadings less than 0.40.

**Automatic functions *(Cronbach’s alpha = 0.78)***	
1.	To avoid or suppress feelings of confusion/aimlessness
2.	To avoid or suppress inner feelings of emptiness
3.	To avoid or suppress negative feelings
4.	To avoid or suppress suicidal thoughts
5.	To avoid or suppress painful images or memories
6.	To obtain a feeling of pleasure
7.	To get into a trance or numb state
**Social functions *(Cronbach’s alpha = 0.83)***	
1.	To show myself how strong I am
2.	To show others how strong I am
3.	To make myself unattractive
4.	To avoid doing chores or tasks I don’t want to do
5.	To avoid being with other people
**Items not included**	
1.	To escape from doing school, work, or other activities
2.	To get attention from others
3.	To punish myself
4.	To provide myself a sense of identity or individuality
5.	To define myself as a person
6.	To escape from a trance or numb state

The Loneliness and Aloneness Scale for Children and Adolescents (LACA; Marcoen, et al., 1987) was used to assess parent- and peer-related loneliness and attitude towards aloneness. The LACA has four subscales (12 items each): parent-related loneliness; peer-related loneliness; affinity towards being alone; and aversion towards being alone. All items are answered on a four-point Likert scale ranging from 1 (*often*) to 4 (*never*). LACA has been validated and used extensively in screening for loneliness in Belgian adolescents (Maes et al., 2015). Cronbach’s alpha for the Loneliness-Parents, Loneliness-Peers, Positive attitude towards aloneness, and Negative attitude towards aloneness subscales in the present were 0.91, 0.89, 0.79, and 0.83 respectively. In line with the literature, correlations among the LACA subscales were low (*r* ranged between –0.10 and 0.39; median *r* = 0.15).

The Beck Depression Inventory II (BDI II; Beck, Steer, & Brown, 1996) was used to assess the degree of depression. The BDI II consists of 21 items that are responded to on a four-point Likert scale ranging from 0 (*not at all*) to 3 (*severely*). The total score for the BDI II can range from 0 to 63 with higher score reflecting more depression (Cronbach’s alpha = .89). The BDI has been extensively used for measuring depression and has been validated in Belgian clinical and non-clinical samples (Vanheule, Desmet, Groenvynck, Rosseel, & Fontaine, 2008).

### Analytical plan

Differences in Loneliness-Parents, Loneliness-Peers, Positive attitude towards aloneness, and Negative attitude towards aloneness as a function of presence vs. absence of NSSI were calculated using the multivariate analysis of covariance (MANCOVA). Gender, age, and depression were added as covariates because they are known to influence NSSI (Xavier, Gouveia, & Cunha, 2016), loneliness, and positive and negative attitudes towards aloneness (Heinrich & Gullone, 2006; Maes et al., 2015). Given that both loneliness and NSSI were strongly related to depression (Nock, 2009; Qualter et al., 2010), we additionally controlled for depression. Pearson correlation coefficients were used to compute the associations between functions of NSSI and the four LACA subscales. To reduce data, the two-factor solution for the functions of NSSI scale, that is, automatic vs. social functions (see also Table 1) suggested by Gandhi et al., (2016) was used.

## Results

In the present sample, lifetime NSSI was found to be 16.5%. Females (*n* = 43) reported a higher lifetime prevalence of NSSI than males (n = 23;\ {\rm{\chi}} _{\left( 1 \right)}^2 = 5.90,\ p = 0.016). The result of the MANCOVA (see Table 2) indicated that when controlling for age and gender, the main effect for lifetime NSSI (Wilk’s λ = 0.866, *F*(4,361) = 13.95, *p* = < 0.001) was statistically significant. Adolescents with lifetime NSSI scored significantly higher on Loneliness-Parents, Loneliness-Peers, and Positive attitude towards aloneness as compared to adolescents without NSSI. When depression was added as an additional covariate in the MANCOVA (see Table 2), the main effect of lifetime NSSI on Loneliness-Parents and Positive-attitude towards aloneness was still statistically significant (Wilk’s λ = 0.966, *F*(4,352) = 3.06, *p* = 0.017).

**Table 2:** A comparison of means (with standard deviations) for lifetime NSSI for the subscales of LACA with covariates.

	NSS1 = 0 (*n* = 301)		NSSI = 1 (*n* = 59)		*F*_1_ (1, 354)	*p*	*F*_2_ (1, 355)	*p*
					
The subscales of LACA	*M*	*(SD)*	*M*	*(SD)*				

Loneliness Parents	17.88	(5.34)	22.69	(8.39)	33.23	<0.001	6.78	<0.001
Loneliness Peers	19.63	(6.07)	24.32	(7.74)	30.60	<0.001	1.99	0.158
Positive attitude towards aloneness	31.42	(5.41)	34.88	(5.53)	19.82	<0.001	4.50	0.035
Negative attitude towards aloneness	29.24	(5.26)	29.31	(5.30)	0.25	0.618	0.32	0.572

*Note: F*_1_ = When controlling for age and gender, *F*_2_ = When controlling for age, gender, and depression.

Findings of the correlation analysis (displayed in Table 3) showed positive correlations between Loneliness-Parents, Loneliness-Peers, and Positive attitude towards aloneness and automatic functions of NSSI. None of the correlations with the social functions were significant.

**Table 3:** Pearson correlations between automatic functions, social functions, and four subscales of LACA. These correlations were calculated only for participants who had engaged in at least one episode of NSSI (*n* = 65).

	Automatic functions		Social functions

Loneliness Parents	0.44	***	0.05
Loneliness Peers	0.46	***	0.17
Positive attitude towards aloneness	0.46	***	0.11
Negative attitude towards aloneness	0.10		0.21

*** *p* < 0.001.

## Discussion

The results of the current exploratory study are largely in agreement with the two earlier mentioned studies (Giletta et al., 2012; Lasgaard et al., 2011). In line with our expectations, individuals engaging in NSSI reported higher levels of parent-related loneliness, even when controlling for age, gender, and depression. However, unlike the previous studies, we found that adolescents with lifetime NSSI also reported higher levels of peer-related loneliness when controlling for gender and age. Mean differences in peer-related NSSI between adolescents with and without NSSI were no longer significant when depression was added as a covariate, suggesting that the relation between peer-related loneliness and NSSI may be mediated by depression (Hayes & Preacher, 2014). Further research is necessary to confirm this hypothesis.

Our findings also indicated that, although a negative attitude towards aloneness did not significantly differ between adolescents with and without NSSI, adolescents with lifetime NSSI reported a higher mean for positive attitude towards aloneness. As previously mentioned, greater need for aloneness in NSSI individuals may be considered as an indirect indicator of maladjustment, because NSSI individuals may use aloneness as a means to avoid contact with others (Goossens & Marcoen, 1999). Adolescents engaging in NSSI may also use time spent on their own to engage in NSSI. Alternatively, as suggested by Goossens (2014), individuals may use aloneness for self-reflection. However, self-reflection may trigger a negative emotional cascade and the individuals may resort to NSSI as a means to regulate these negative emotions (Selby, Connell, & Joiner, 2010). Another possible reason for the observed correlation between positive attitude towards aloneness and NSSI may be attributed to the conceptualization of positive attitude towards aloneness in the LACA, the loneliness scale used in the current study.

The results of the correlational analysis further clarified the relations between loneliness, attitude towards aloneness, and NSSI functions. The positive correlation between parent- and peers-loneliness and the automatic function of NSSI can be explained by the fact that loneliness is an internal affective state associated with the evaluation of one’s social relationships (Heinrich & Gullone, 2006). Our results indicate that lonely adolescents may use NSSI as a means of managing distress associated with loneliness and not as a means of managing interpersonal relations and expectations (at least partially confirming the findings of Nock & Prinstein, 2005). Additionally, we found a positive association between positive attitude towards aloneness and automatic functions of NSSI. In line with Goossens and Marcoen, (1999), we hypothesize that adolescents with positive attitude towards aloneness tend to be alone as they want to avoid social contact because of their NSSI. Further research is necessary to confirm this hypothesis.

The readers should note that the association between loneliness and NSSI is likely to be bidirectional. Chronic loneliness can increase the vulnerability to NSSI by influencing both body physiology and psychological processes. Physiologically, chronic loneliness can lead to a prolonged activation of the Hypothalamo-Pituitary-Adrenal (HPA) axis, which in turn may lead to consistently high levels of cortisol in the body (Hawkley & Cacioppo, 2010). Persistently high levels of cortisol may lead to aberrations in genome-wide DNA methylation which has been shown to increased depression, anxiety, chronic fatigue, and cognitive impairment (Glad et al., 2017), and ultimately may also increase vulnerability to NSSI. Psychologically, loneliness can disrupt self-regulatory mechanisms. More specifically, individuals may engage in behaviors in which they would otherwise never engage in just to alleviate the negative affect associated with loneliness (Crepaz & Marks, 2001). On the other hand, engagement in NSSI may lead to feelings of shame, guilt, and regret that can increase social isolation. In addition to the affective consequences, the physical consequences of NSSI (e.g., scaring) along with the fear of stigmatization can also lead to further social isolation (Garisch & Wilson, 2015).

In spite of extending the literature on NSSI, the present work is not without its limitations. First, because of the cross-sectional nature of the study, no conclusions can be drawn regarding the directionality of effect between the study variables. As mentioned above, the relation between NSSI, loneliness, and attitude towards aloneness are likely to be bi-directional. Second, our findings cannot be extended beyond the current sample as it was based on convenience sampling. Further research with a larger and a representative sample may be necessary to confirm our findings. Third, although self-report measures are appropriate for measuring loneliness and attitude towards aloneness, shared method variance may have led to inflated correlations among the study variables.

Overall, the current study not only highlighted the importance of two developmentally relevant constructs, that is, loneliness and attitude towards aloneness in the context of NSSI, but it also suggested possible mechanisms connecting these constructs. Further longitudinal research is necessary to test these hypotheses more conclusively.
